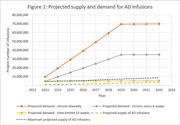# Is the US infusion capacity sufficient for the expected demand with the emergence of disease‐modifying Alzheimer’s treatments?

**DOI:** 10.1002/alz.087651

**Published:** 2025-01-09

**Authors:** Soeren Mattke, Jiahe Chen, Mark Hanson

**Affiliations:** ^1^ University of Southern California, Los Angeles, CA USA; ^2^ University Of Southern California, Los Angeles, CA USA

## Abstract

**Background:**

Disease‐modifying treatments for Alzheimer’s disease (AD) are becoming available. To date, all current treatments require intravenous infusion, and concerns have emerged that lack of infusion capacity may limit access to treatment. We predict supply and demand for infusions for AD treatment in the US under different assumptions over a 10‐year period.

**Method:**

We use a Markov model to predict the number of individuals identified as eligible for AD treatment from 2023 to 2032, taking into account constrained capacity for AD specialist visits and confirmatory biomarker testing. We analyze chronic treatment starting at mild cognitive impairment (70% of patients) and mild dementia (30%) until transition to moderate dementia with infusions every two and four weeks for an estimated duration of 5.7 years, and time‐limited treatment every four weeks for 52 weeks as a lower bound estimate. National historic trends of infusion capacity were obtained from the CDC’s Ambulatory Health Care Data surveys and projected forward. Estimates for excess capacity and future growth of capacity for AD treatment were obtained from a survey covering 404 US infusion centers.

**Result:**

The model predicts that 370,527 patients would be eligible to start treatment in 2023, increasing by 1% per year. Based on the CDC data, around 21 million infusions across therapeutic areas would be provided in 2023 with a projected annual growth rate of 3%. The survey of infusion centers suggests that 3% of capacity is currently devoted to AD with that share growing 1% per year, as well as up to 17% average unused capacity. Figure 1 shows the projected supply and demand. The solid lines show demand for the three types of treatment, the green and black dashed lines the projected supply and the maximum projected supply, if all unused capacity were devoted to AD infusions.

**Conclusion:**

Projected US infusion capacity would be insufficient for all three treatment types, and even the maximum projected capacity would only be sufficient for time‐limited treatments. Expansion of capacity and introduction of treatments that do not require intravenous infusions will be necessary to ensure timely access to treatment.